# Myeloid Clonal Infiltrate Identified With Next-Generation Sequencing in Skin Lesions Associated With Myelodysplastic Syndromes and Chronic Myelomonocytic Leukemia: A Case Series

**DOI:** 10.3389/fimmu.2021.715053

**Published:** 2021-10-04

**Authors:** Grégoire Martin de Frémont, Pierre Hirsch, Santiago Gimenez de Mestral, Philippe Moguelet, Yoan Ditchi, Jean-François Emile, Patricia Senet, Sophie Georgin-Lavialle, Thomas Hanslik, François Maurier, Amir Adedjouma, Noémie Abisror, Thibault Mahevas, Florent Malard, Lionel Adès, Pierre Fenaux, Olivier Fain, François Chasset, Arsène Mekinian

**Affiliations:** ^1^ Sorbonne Université, Assistance Publique-Hôpitaux de Paris (AP-HP), Hôpital Saint-Antoine, Service de Médecine Interne et Inflammation-[Département Médico-Universitaire (DMU)-i3], Université Paris 06, Paris, France; ^2^ Sorbonne Université, INSERM, Centre de Recherche Saint-Antoine, AP-HP, Hôpital Saint-Antoine, Service d’Hématologie Biologique, Université Paris 06, Paris, France; ^3^ Sorbonne Université, AP-HP, Hôpital Saint-Antoine, Service d’Anatomopathologie, Université Paris 06, Paris, France; ^4^ Service d’Anatomopathologie, Hôpital Ambroise Paré, Assistance Publique Hôpitaux de Paris (APHP) and Université de Versailles Saint Quentin en Yvelines, Boulogne Billancourt, France; ^5^ Sorbonne Université, AP-HP, Hôpital Tenon, Service d’Anatomopathologie, Université Paris 06, Paris, France; ^6^ Sorbonne Université, AP-HP, Hôpital Tenon, Service de Dermatologie, Université Paris 06, Paris, France; ^7^ Sorbonne Université, AP-HP, Hôpital Tenon, Service de Médecine Interne, Université Paris 06, Paris, France; ^8^ Service de Médecine Interne, Hôpital Ambroise Paré, Assistance Publique Hôpitaux de Paris (APHP) and Université de Versailles Saint Quentin en Yvelines, Boulogne Billancourt, France; ^9^ Service de Médecine Interne et Immunologie Clinique, Groupe Hospitalier UNEOS, Metz, France; ^10^ Sorbonne Université, AP-HP, Hôpital Saint-Antoine, Service d’Hématologie Clinique, Université Paris 06, Paris, France; ^11^ Service d’Hématologie-Sénior, Hôpital Saint-Louis, Assistance Publique Hôpitaux de Paris (APHP) and Université de Paris, Paris, France

**Keywords:** myelodysplastic syndrome, chronic myelomonocytic leukemia, skin, next-generation sequencing, clonal hematopoiesis

## Abstract

**Background:**

Myelodysplastic syndromes (MDS) and chronic myelomonocytic leukemia (CMML) are associated with cutaneous manifestations. Next-generation sequencing (NGS) is a tool capable of identifying clonal myeloid cells in the skin infiltrate and thus better characterize the link between hematological diseases and skin lesions.

**Objective:**

To assess whether skin lesions of MDS/CMML are clonally related to blood or bone marrow cells using NGS.

**Methods:**

Comparisons of blood or bone marrow and skin samples NGS findings from patients presenting with MDS/CMML and skin lesions in three French hospitals.

**Results:**

Among the 14 patients recruited, 12 patients (86%) had mutations in the skin lesions biopsied, 12 patients (86%) had a globally similar mutational profile between blood/bone marrow and skin, and 10 patients (71%) had mutations with a high variant allele frequency (>10%) found in the myeloid skin infiltrate. Mutations in *TET2* and *DNMT3A*, both in four patients, were the most frequent. Two patients harbored a *UBA1* mutation on hematopoietic samples.

**Limitations:**

Limited number of patients and retrospective collection of the data. Blood and skin sampling were not performed at the exact same time point for two patients.

**Conclusion:**

Skin lesions in the setting of MDS/CMML are characterized by a clonal myeloid infiltrate in most cases.

## Introduction

Myelodysplastic syndromes (MDS) are defined as clonal hematopoietic disorders with ineffective hematopoiesis resulting in dysplasia, bone marrow failure, and peripheral cytopenias. Chronic myelomonocytic leukemia (CMML) is closely related to MDS and characterized by persistent monocytosis with medullar dysplastic features (1). During the last years, the diagnosis, prognosis staging, and therapeutical management of MDS/CMML have been modified by the emergence of next-generation sequencing (NGS), which allows the screening of mutations in multiple genes. Blood and/or bone marrow NGS allows to identify somatic mutations in more than 90% of patients suffering from MDS/CMML (2). In the setting of MDS/CMML, systemic inflammatory and autoimmune diseases (SIADs) occur in 10 to 30% patients, mostly neutrophilic diseases, connective tissue disorders, arthritis, and vasculitis (3–7). More than half of patients with SIADs have skin involvement, consisting in neutrophilic dermatosis and skin vasculitis, less frequently cutaneous granulomatosis (8–10). Other skin lesions including cutaneous infections, drug adverse reactions, and *leukemia cutis* (blastic myeloid cell infiltration of the skin) can be observed and must be distinguished from SIADs (11). Moreover, using fluorescent *in situ* hybridization (FISH) analyses, some studies suggested the skin infiltrate of neutrophilic dermatoses that arise in the context of myeloid malignancies have differentiated from the malignant clone (12). This has been shown particularly for histiocytoid Sweet’s syndrome (H-SS) (13). Similarly, a recent study using NGS demonstrated a clonal relationship between malignant cells of myeloid neoplasm and neutrophil infiltrate of Sweet’s syndrome (14). However, these findings were restricted to neutrophilic dermatoses and included a limited number of MDS/CMML patients. Interestingly, a case of NGS identifying a clonal infiltrate in *myelodysplasia cutis*, consisting in an immature myeloid histiocytoid non-blastic infiltrate in the setting of MDS, was recently reported, as defined by Osio et al. (15, 16). Given these preliminary data, we sought to better characterize skin lesions of patients with MDS/CMML using NGS, hypothesizing that a majority of skin lesions could be related to a clonal myeloid infiltrate of the skin and thus share common mutations with the hematological MDS/CMML clone found in blood and marrow.

## Patients and Methods

### Patients

All consecutive patients with MDS or CMML referred to our department of internal medicine or dermatology in university tertiary centers between 2015 and 2020 for skin eruptions were enrolled in the study if a skin biopsy and NGS on at least one skin specimen were available. Skin lesions with a specific myelomonocytic infiltrate in pathology were referred as CMML *cutis*. Histiocytoid Sweet Syndrome (H-SS) was defined as a dermal infiltrate composed mostly of immature myeloid cells with histiocytic appearance as previously described (17). We decided to use the term *myelodysplasia cutis* only if lesions strictly corresponding to Osio et al.’s clinical and histological description were sampled (16). CMML/Erdheim Chester disease (ECD) was defined as a clonal cutaneous CMML infiltrate fulfilling ECD criteria (18). Of note, some biopsies did not have extensive immunohistochemical studies. *Leukemia cutis* was defined as a blastic infiltrate with CD34+, CD117+, or CD56+ cells. Skin biopsies of other patients addressed to our hospitals for *leukemia cutis* also underwent NGS to compare mutations and variant allele frequency (VAF) with those of non-blastic skin lesions. Three patients included in our case-series had previously been reported (18, 19).

### Molecular Analysis

DNA was extracted from total bone marrow or blood samples using QIAsymphony (Qiagen) method, and from fresh or formalin-fixed paraffin-embedded (FFPE) tissue for skin samples (18). For a majority of samples, libraries were obtained from 112.5 ng of DNA using HaloPlex Target Enrichment System^®^ (Agilent technologies), using a gene panel including main genes implied in myeloid malignancies as already described (20). For three samples (patients 9, 13, and 14) without enough DNA sample available for a second technique, other similar gene panels designed for myeloid malignancies diagnosis were used. Libraries were sequenced on a MiSeq^®^ sequencer (IlluminaINC). Read alignment, variant calling, and annotation were performed using Sophia DDM^®^ software version 5.0.12 (Sophia genetics). The sensitivity was 1%. All variants were checked using Integrative Genomics Viewer (IGV) software v2.3.

Sanger sequencing was performed for *UBA1* gene exon 3 for 12 patients with available hematopoietic samples (all patients but 8 and 12) and for one patient on skin sample (patient 8), as it was not included in the original gene panel.

### Ethics

The study was conducted according to the French CNIL methodology reference and in accordance with the Declaration of Helsinki. Written informed consent was obtained from the patients unless dead at the time of the study.

## Results

### Patients’ Characteristics

The patients are described in **Tables 1**, **2**. The median age of the 14 patients was 70 years old at MDS/CMML diagnosis (ranging 62–81) and was 69 years old at the time of cutaneous manifestations (ranging 30–82). The MDS subtypes were MDS with multilineage dysplasia (n=3, including one with myelofibrosis), MDS with excess blasts (n=1), MDS with unilineage dysplasia (n=1), MDS/myeloproliferative neoplasm (MPN) (n=2), and CMML (n=7). Karyotype was normal in eight patients, abnormalities were found in five patients [del(20)(q11q13) in patient 5; iso(X)(p10) and t(3,12)(p2?2;q24) in patient 6; trisomy 18 in patient 11; trisomy 8, del(1)(p?3?4), and del(5) (q?3?1q35) in patient 13; and del(4)(q?23q?26) in patient 14], and the karyotype could not be performed for one patient (patient 7). The MDS were low or intermediate-1 risk in 93% cases with median IPSS at 0.5 (0–2) and very-low, low, or intermediate risk in 93% cases with median IPSS-R at 2.3 (0.6–5.1).

**Table 1:** Patients and skin lesion characteristics.

Patients’ characteristics (n = 14)	
Male n (%)	8 (57%)
Median age at MDS/CMML diagnosis, years old (range)	70 (62–81)
Median age at skin lesions diagnosis, years old (range)	69 (30–82)
Skin lesions preceding MDS/CMML n (%)	5 (36%)
MDS subtypes:	
MDS-MLD (%)	3 (22%)
MDS-ULD (%)	1 (7%)
MDS-EB (%)	1 (7%)
MDS/MPN (%)	2 (14%)
CMML (%)	7 (50%)
Abnormal karyotype (%)	5 (36%)
IPSS n (%):	
Low risk	7 (50%)
Intermediate-1	6 (43%)
Intermediate-2	1 (7%)
High	0
Extracutaneous manifestations n (%):	7 (50%)
Fever (%)	5 (36%)
Arthralgia or arthritis (%)	6 (43%)
**Skin lesion characteristics**	
Total number of biopsies	14
Diagnosis after clinic-pathological correlation:	
H-SS	4
CMML *cutis*	3
Erdheim Chester disease/CMML *cutis*	2
Kikuchi-Fujimoto lupus	1
Neutrophilic folliculitis	1
Erythema nodosum	1
Pyoderma gangrenosum	1
Livedo reticularis with inconclusive skin biopsy	1
**Next-generation sequencing**	
Positive NGS on hematopoietic samples	14 (100%)
Number of mutations, median (range)	2 (1–8)
Positive NGS on skin biopsies	12 (86%)
Percentage of myeloid cells, median (range)	20% (5–>50%)
Number of mutations, median (range)	3.5 (1–7)
Diagnosis of skin clonal infiltrate following NGS	12 (86%)
*Among positive NGS in skin biopsies:*	
Same main mutations in both tissues	8 (67%)
Patients with at least one mutation in skin absent from blood/BM	4 (33%)
Highest VAF>10%	10 (83%)
Including VAF>20%	6 (50%)
Most frequent mutations:	
*TET2*	4 (33%)
*DNMT3A*	4 (33%)

CMML, chronic myelomonocytic leukemia; EB, excess blasts; ECD, Erdheim-Chester disease; IPSS, international prognostic scoring system; MDS, myelodysplastic syndromes; MLD, multi-lineage dysplasia; MPN, myeloproliferative neoplasm; NGS, next-generation sequencing; (H)-SS, (Histiocytoid-)Sweet’s syndrome; ULD, uni-lineage dysplasia; VAF, variant allele frequency.

**Table 2:** Detailed patients’ characteristics.

ID	Age at first cutaneous lesions (years)	Age at hematological malignancy diagnosis (years)	Sex	Hematological malignancy	Cytogenetics	Skin lesions	Main clinical hypothesis/es before biopsy	Histology of skin biopsy	Immuno-histochemistry of skin biopsy	Diagnosis after clinic-pathological correlation	Skin clonal mutations (Yes/No)	VEXAS syndrome	Extracutaneous manifestations
**1**	62	62	F	CMML	N	Periorbital and circumferential xanthomatous lesions	Xanthelasma associated with histiocytosis	Dense histiocytic infiltrate with plurinucleate cells and Touton cells	CD68+, CD163+, CD1a−	ECD/CMML *cutis*	Yes	No	No
**2**	71	71	M	MDS-MLD and myelofibrosis	N	Papulonodular lesions of the limbs and abdomen	Vasculitis or HSS	Dermal plurinucleate infiltrate, partly immature	MPO+	H-SS	Yes	Yes	Fever, asthenia, arthritis, panuveitis
**3**	54	65	F	CMML	N	Periorbital and circumferential xanthomatous lesions	Xanthelasma associated with histiocytosis	Dense histiocytic infiltrate with plurinucleate cells and Touton cells	CD68+, CD163+, CD1a−	ECD/CMML *cutis*	Yes	No	Asthenia, arthritis, pleuro-pericarditis, splenic infarct
**4**	72	70	M	CMML	N	Erythemato-squamous papulonodular lesions of the limbs and back	CMML *cutis*	Dermal lymphohistiocytic infiltrate with plurinucleate cells	NA	CMML *cutis*	Yes	No	No
**5**	61	61	F	MDS-ULD	Del(20)(q11q13)	Diffuse, painful, and sometimes erosive erythematous and violaceous papulonodular skin lesions of the extremities, thighs, arms, breasts, ears, and nose	Tumid lupus *or* SS *or* Kikuchi-Fujimoto lupus	Dermal inflammatory infiltrates with mainly regular mononuclear cells without dysplasia (lymphocytes, macrophages, plasmacytoid dendritic cells), nuclear debris or neutrophils associated with vacuolar alteration of basal keratinocytes, deep perivascular infiltrate of lymphocytes, and abundant mucin deposits in the reticular dermis	MPO+, CD163+, CD123+	Kikuchi-Fujimoto lupus	No	No	No
**6**	82	75	F	CMML	Iso(X)(p10), t(3;12)(p2?2;q24)	Erythematous and pruriginous papular lesions of the head, limbs, and trunk	CMML *cutis or* eczema	Dermal lymphoid and immature myeloid infiltrate	MPO+, CD68+, CD163+	CMML *cutis*	Yes	No	No
**7**	69	69	F	MDS-MLD	Too few mitoses to be performed	Diffuse erythematous papular lesions	SS *or leukemia cutis*	Dermal lymphohistiocytic infiltrate	NA	H-SS	Yes	No	No
**8**	NA	70	F	MDS-EB2	N	Livedo	Livedo associated with thrombophilia	No abnormalities	NA	Inconclusive biopsy and no underlying thrombophilia	No*	No	Fever, asthenia, pulmonary embolism, 8 spontaneous miscarriages
**9**	69	68	M	MDS-EB2/MPN	N	Erythematous maculopapular lesions of the limbs and trunk	SS	Dermal lymphoid infiltrate with plurinucleate cells	CD163+	H-SS	Yes	No	No
**10**	82	81	M	MDS-ULD/MPN	N	Pustules of the scalp	Folliculitis	Dermal lymphohistiocytic infiltrate with neutrophils cells	CD163+	Neutrophilic folliculitis	Yes	No	Sjogren’s syndrome (sicca syndrome, arthritis)
**11**	80	81	M	CMML	Trisomy 18	Erythematous papulonodular lesions	SS *or Leukemia cutis*	Dermal infiltrate of eosinophilic myelomonocytic cells	MPO+, CD68+, CD163, CD45+, CD56+	CMML *cutis*	Yes	No	Adult Still disease (fever, asthenia, arthritis, pleuro-pericarditis)
**12**	69	71	M	MDS-MLD	N	Papulopustular lesions	SS *or* infectious	Dermal histiocytic infiltrate with plurinucleate cells	CD34+, CD163+	H-SS	Yes	Yes	Kikuchi-Fujimoto lupus (fever, asthenia, arthritis)
**13**	77	77	M	CMML	Trisomy 8, del(1)(p?3?4), del(5)(q?3?1q35)	Nodular lesions of the lower limbs	SS *or* erythema nodosum	Hypodermal lymphohistiocytic infiltrate with few plurinucleate cells with an aspect of septal panniculitis	NA	Erythema nodosum	Yes	NA	Fever, asthenia, arthritis, digestive vasculitis
**14**	31	68	M	CMML	Del(4)(q?23q?26)	Violaceous ulcerated and pustular lesions	Pyoderma gangrenosum	Dense neutrophil infiltrate with histiocytes	NA	Pyoderma gangrenosum	Yes	No	No

CMML, chronic myelomonocytic leukemia; del, deletion; EB2, excess blasts subtype 2; ECD, Erdheim-Chester disease; F, female; iso, isochromosome; M, male; MDS, myelodysplastic syndromes; MLD, multilineage dysplasia; MPN, myeloproliferative neoplasm; MPO, myeloperoxidase; N, normal; NA, not available; (H)-SS, (Histiocytoid-)Sweet’s syndrome; t, translocation; ULD, unilineage dysplasia; ‑, biopsied skin lesions; ±, skin biopsy analyzed with NGS; *, the 3% skin VAF with 0% myeloid infiltrate was interpretated as a blood contamination facilitated by a highly circulating clone (blood VAF 80%).

Skin lesions preceded the MDS/CMML diagnosis in 5 (36%) patients. In 7 (50%) cases, extracutaneous symptoms were associated; fever (n=5; 36%) and arthralgia and/or arthritis (n=6; 43%) were the most prevalent.

### Cutaneous and Histological Features of MDS/CMML Patients

**Figure 1:**
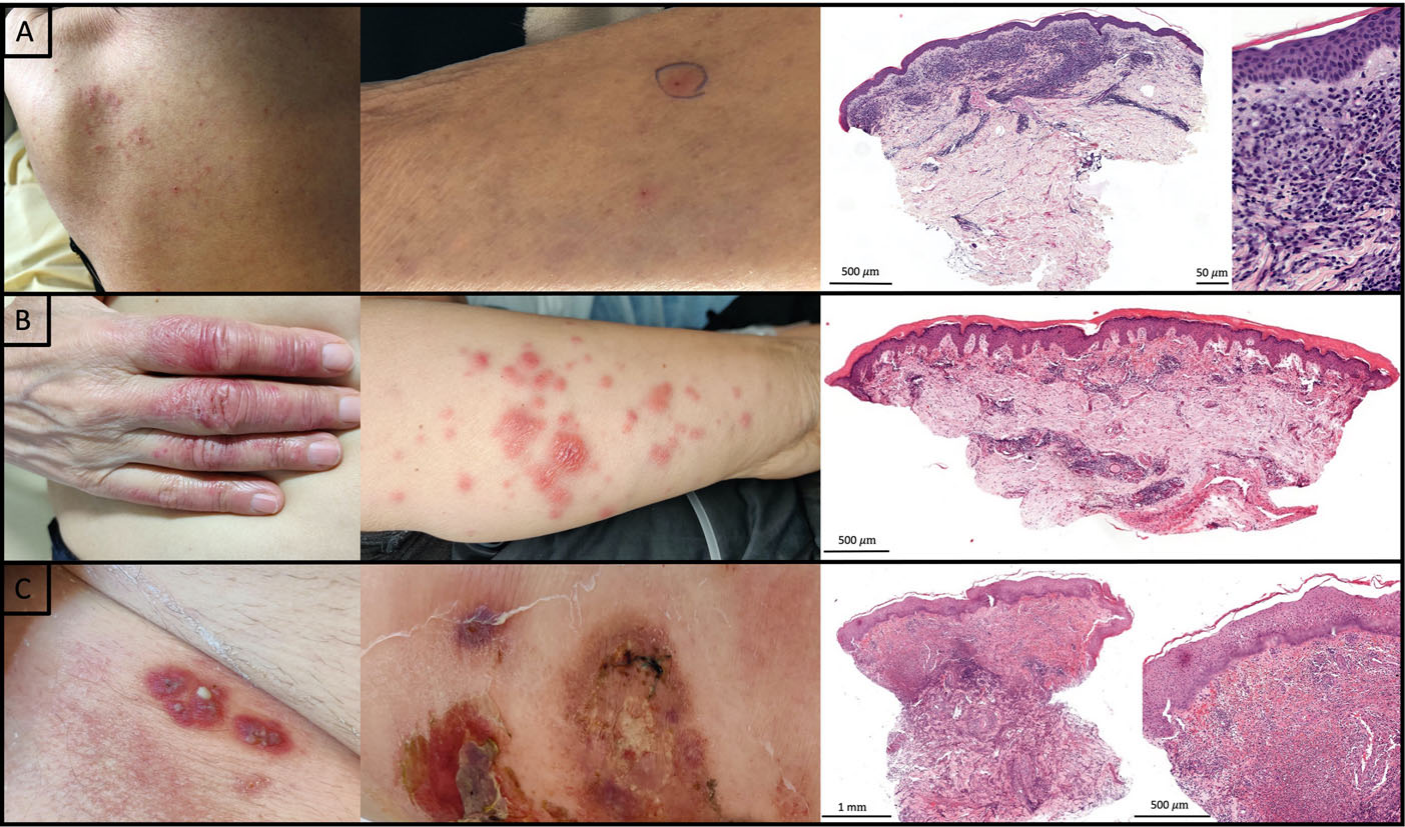
Clinical and histological features of patient 6, 7, and 14. **(A)** Patient 6, from left to right: Erythematous excoriated papules of the back; Erythematous crusty papule of the arm in which was performed skin biopsy; Dense superficial dermal infiltrate of myeloid cells (CD68+ monocytes and rare MPO+ cells) associated with lymphocytes compatible with CMML *cutis* (Hematoxylin-eosin-saffron; Original magnification respectively ×30 and ×400). **(B)** Patient 7, from left to right: papulonodular lesions of the hand and forearm; Dermal lymphohistiocytic infiltrate (Hematoxylin-eosin-saffron; Original magnification ×25). **(C)** Patient 14, from left to right: violaceous pustular and ulcerated lesions; Dense dermal neutrophilic infiltrate with histiocytes (Hematoxylin-eosin-saffron; Original magnification respectively ×25 and ×150).

Based on clinical, histological, and immunohistochemical findings and before NGS results, skin lesions have been classified as H-SS in four cases (patients 2, 7, 9, and 12), *CMML cutis* in three cases (patients 4, 6, and 11), and ECD associated with CMML *cutis* in two cases (patients 1 and 3) (18), Kikuchi-Fujimoto disease in one case (patient 5), neutrophilic folliculitis in one case (patient 10), erythema nodosum in one case (patient 13), pyoderma gangrenosum in one case (patient 14), and extensive livedo reticularis with non-specific findings on skin biopsy in one case (patient 8) (**Tables 1**, **2**). Clinical and histological lesions are shown for patients 6, 7, and 14 (**Figure 1**).

### Comparison of Blood/Bone Marrow and Skin NGS Results

Patients all had concomitant blood/marrow and skin sampling apart from patient 2 (skin biopsy performed 1 year before blood analysis with NGS) and patient 13 (skin biopsy performed 2 years before bone marrow analysis with NGS). At least one mutation was identified using NGS in blood/bone marrow in all patients with a median number of two mutations (ranging 1–8) and in skin biopsies in 12 patients with a median number of 3.5 mutations (ranging 1–7). Patient 5 was considered negative on skin owing to a very low skin VAF (<1%). Patient 8 was considered as a blood contamination as the weakly positive skin NGS (3% VAF) was not associated with a cutaneous myeloid infiltrate. Median VAF in skin was 12% (ranging from 1 to 76%) (**Table 1**). The same main mutation between blood/bone marrow and skin was found in eight patients (67% of patients with mutations in the skin). Six patients had paired mutations in blood/marrow and skin lesions (patients 1, 2, 3, 4, 11, 12 with respectively ECD/CMML *cutis*, H-SS, ECCD/CMML *cutis*, CMML *cutis*, CMML *cutis*, and H-SS). For the six other patients, three had more mutations on blood/marrow than skin (patients 6, 10, and 14, with respectively CMML *cutis*, neutrophilic folliculitis, and pyoderma gangrenosum), and three had more mutations on skin than blood/marrow (patients 7, 9, and 13, with respectively H-SS, H-SS, and erythema nodosum). Patient 7 had one extra-mutation in skin (in *TP53*, VAF 2%), patient 9 had two extra-mutations in skin (in *SETBP1*, VAF 4%, and in *KMT2D/MLL2*, VAF 3%), and patient 13 had two extra-mutations in skin (in *KRAS*, VAF 11%, and in *RUNX1*, VAF 6%) and one extra-mutation in blood (in *ZRSR2*, VAF 7%) with a bone marrow analysis performed 2 years after skin sampling (**Tables 2**, **3**). Regarding VAF, 10 patients had at least one mutation in skin with VAF >10% (patients 1, 3, 4, 6, 7, 9, 10, 11, 13, 14, with respectively ECD/CMML *cutis*, ECD/CMML *cutis*, CMML *cutis*, CMML *cutis*, H-SS, H-SS, neutrophilic folliculitis, CMML *cutis*, erythema nodosum, and pyoderma gangrenosum), of whom six had at least one mutation with VAF >20% (patients 4, 7, 10, 11, 13, 14, with respectively CMML *cutis*, H-SS, neutrophilic folliculitis, CMML *cutis*, erythema nodosum, and pyoderma gangrenosum), and two patients had detectable mutation with VAF <10% (patients 2 and 12 with 40 and 20% myeloid cells in the infiltrate, respectively, both diagnosed with H-SS). The myeloid infiltrate was of only 5% in three patients, but all of them had VAF >10% (patients 4, 7, and 9) (**Tables 2**, **3**). The most frequent mutations were in *DNMT3A* and *TET2* both found in four patients. Two patients harbored the recently identified mutations in *UBA1* (2 and 12) (21), both having H-SS associated with a febrile polyarthritis.

**Table 3:** Mutations in hematopoietic and skin samples.

ID	Percentage of myeloid cells on the skin infiltrate	Gene(s)	Mutation	VAF hematopoietic sample (%)	VAF skin biopsy (%)
**1**	30%	***NRAS***	NM_002524:c.38G>A:p.Gly13As	41%	16%
**2**	40%	***ZRSR2***	NM_005089:c.112dupA:p.Arg38Lysfs	17%	2%
		***SF3B1***	NM_012433:c.2242A>G:p.Lys748Glu	6%	2%
		***UBA1^†^***	NM_153280.3:c.122T>C:p.Met41Thr	Yes	NA
**3**	40%	***KRAS***	NM_004985:c.35G>A:p.Gly12Asp	46%	19%
		***ASXL1***	NM_015338:c.1934dupG:p.Gly646Trpfs	42%	15%
**4**	5%	***TET2***	NM_001127208:c.822delC:p.Asn275IIefs*	54%	14%
		***DNMT3A***	NM_175629:c.1803G>A:p.Trp601*	46%	21%
**5**	10%	***DNMT3A***	NM_175629:c.2644C>T:p.Arg882Cys	2%	<1%
**6**	20%	***DNMT3A***	NM_175629:c.1917_1918delCT:p.Phe640fs*	33%	11%
		***RUNX1***	NM_001001890:c.877C>T:p.Arg293*	30%	10%
		***SRSF2***	NM_003016:c.284C>A:p.Pro95His	28%	14%
		***TET2***	NM_001127208:c.1732delC:p.His578IIefs*	26%	14%
		***CBL***	NM_005188:c.870-1G>A:p.?	3%	Not detected
**7**	5%	***TP53***	NM_001126114:c.734G>A:p.Gly245Asp	19%	21%
		***TP53***	NM_001126114:c.824G>C:p.Cys275Ser	15%	25%
		***TP53***	NM_001126114:c.101-1G>A:p.?	Not detected	2%
**8**	0%	***TET2***	NM_001127208:c.1061delC:p.Ser354*	80%	3%
		***SRSF2***	NM_003016:c.284C>A:p.Pro95His	24%	Not detected
**9**	5%	***SRSF2***	NM_003016:c.284C>G:p.Pro95Arg	35%	11%
		***EP300***	NM_001429:c.2104delA:p.Met702*	30%	7%
		***CUX1***	NM_001202543:c.1316dupC:p.Ala439fs	7%	11%
		***ASLX2***	NM_018263:c.1471C>T:p.Gln491*	5%	9%
		***SETBP1***	NM_015559:c.2608G>A:p.Gly870Ser	Not detected	4%
		***KMT2D/MLL2***	NM_001130442:c.11911_11912insTTC:p.Gln3971delinsLeuGln	Not detected	3%
**10**	40%	***EZH2***	NM_004456:c.394C>G:p.Pro132Ala	75%	36%
		***ASXL1***	NM_015338:c.1934dupG:p.Gly646Trpfs	48%	22%
		***IDH2***	NM_001289910:c.419C>G:p.Arg140Gln	46%	26%
		***RUNX1***	NM_001001890:c.871dupT:p.Ser291Phefs	31%	13%
		***EZH2***	NM_004456:c.2071T>G:p.Phe691Val	6%	Not detected
		***RUNX1***	NM_001001890:c.904_908del:p.Ala302Glnfs	2%	Not detected
		***RUNX1***	NM_001001890:c.1046_1047del:p.Arg349Leufs	2%	Not detected
**11**	>50%	***SMC1A***	NM_6306:c.1757G>A:p.Arg586Gln	95%	76%
		***ASXL1***	NM_015338:c.1720-1G>T:p.?	51%	40%
		***EZH2***	NM_004456:c.1973A>C:p.Tyr658Ser	50%	40%
		***SETBP1***	NM_015559:c.2608G>A: p.Gly870Ser	50%	38%
**12**	20%	***TET2***	NM_001127208:c.3384T>G:p.Tyr1128	4%	1%
		***DNMT3A***	NM_175629:c.2695C>G:p.Arg899Gly	1%	2%
		***UBA1^†^***	NM_153280.3:c.121A>C:p.Met41Leu	Yes	NA
**13**	20%	***ASXL1***	c.2338C>T:p.Gln780	34%	29%
		***RIT1***	c.246T>A:p.Phe82Leu	33%	7%
		***ZRSR2***	c.212T>A :p.Leu71	7%	Not detected
		***KRAS***	c.173C>T:p.Thr58IIe	Not detected	11%
		***RUNX1***	c.593A>Gly:p.Asp198Gly	Not detected	6%
**14**	>50%	***TET2***	NM_001127208:c.5676delT: p.His1893Thrfs*15	48%	64%
		***DNMT3A***	NM_175629:c.1154delC: p.Pro385Argfs*22	47%	48%
		***PPM1D***	NM_003620.3:c.1535dupA: p.Asn512Lysfs*16	30%	Not detected
		***TP53***	NM_001126114:c.613T>A: p.Tyr205Asn	26%	19%
		***TET2***	NM_001127208:c.4035T>A: p.Tyr1345*	11%	1%
		***TET2***	NM_001127208:c.4447G>T: p.Glu1483*	4%	4%
		***TET2***	NM_001127208:c.4210C>T: p.Arg1404*	1%	1%
		***U2AF1***	NM_007279.2:c.1009C>T: p.Gln157Pro	1%	8%

*Next-generation sequencing for all mutations shown apart from ^†^UBA1 was sequenced with Sanger’s technique.

### Outcome and Treatment

Eleven patients have been treated and received oral steroids (n=8), hydroxychloroquine (n=2), immunosuppressive therapies (dapsone n=1), biological-targeted therapy (anakinra n=2, low-dose interleukin-2 n=1), or azacytidine as the hematological therapy (n=7). Azacytidine was associated with complete skin disease remission in three patients (two H-SS and one CMML *cutis*), partial in two (H-SS and pyoderma gangrenosum), and did not improve skin lesions in two (xanthelasma and folliculitis) (**Table 4**).

**Table 4:** Treatment outcome in skin lesions associated to MDS/CMML.

ID	Skin lesions	Mutations in the skin infiltrate	Systemic treatments received	Efficacy on skin lesions
1	ECD/CMML *cutis*	*NRAS*	None	–
2*	H-SS	*ZRSR2, SF3B1*	Oral steroids	Partial (steroid dependence)
3	ECD/CMML *cutis*	*KRAS, ASXL1*	Oral steroids	None
			Hydroxychloroquine	
			Azacytidine	
4	CMML *cutis*	*TET2, DNMT3A*	None	–
5	Kikuchi-Fujimoto lupus	*None*	Oral steroids Hydroxychloroquine	Partial
6	CMML *cutis*	*DNMT3A, RUNX1, SRSF2, TET2*	Oral steroids Azacytidine	Total
7	H-SS	*TP53, TP53, TP53*	Azacytidine	Total
8	Inconclusive biopsy and no underlying thrombophilia	*None*	None	–
9	H-SS	*SRSF2, EP300, CUX1, ASXL2, SETBP1, KMT2D/MLL2*	Oral steroids	Total
			Azacytidine	
10	Neutrophilic folliculitis	*EZH2, ASXL1, IDH2, RUNX1*	Oral steroids	None
			Low dose IL-2	
			Azacytidine	
11	CMML *cutis*	*SMC1A, ASXL1, EZH2, SETBP1*	Anakinra	Total
12*	H-SS	*TET2, DNMT3A*	Anakinra	Partial
			Azacytidine	
13	Erythema nodosum	*ASXL1, RIT1, KRAS, RUNX1*	Oral steroids	Total
14	Pyoderma gangrenosum	*TET2, DNMT3A, TP53, TET2, TET2, TET2, U2AF1*	Oral steroids	Partial
			Dapsone	
			Azacytidine	

CMML, chronic myelomonocytic leukemia; ECD, Erdheim-Chester disease; H-SS, histiocytoid Sweet’s syndrome; IL, interleukin.

*Patients with VEXAS syndrome.

Two out of the 14 patients were dead by the end of data collection. One of them died of central nervous system infection (patient 11), and the other one of acute myeloid leukemia (patient 13).

### Comparison to Patients With *Leukemia Cutis*

It is known that *leukemia cutis* is characterized by a skin infiltrate of blastic cells directly related to the myeloid leukemia. Therefore, NGS mutational profile of blood and skin lesions should be similar, and these patients could be used as “positive controls.” We aimed to compare the results of our MDS/CMML patients with patients having *leukemia cutis* focusing on the “VAF/percentage of myeloid cells in the skin infiltrate” ratio. Four patients referred for cutaneous lesions compatible with *leukemia cutis* had NGS on skin samples. Two had AML and two had CMML evolving to AML. Skin symptoms were prominent with nodular lesions diagnosed in three patients and erythroderma in one. *Leukemia cutis* NGS found a median number of mutations of 4 (*versus* 4,5 in blood or marrow for the same patients). The median VAF was of 22% (ranging from 2 to 71%) in *leukemia cutis* (with a median percentage of myeloid cells in the infiltrate of >50%) compared with 12% (ranging from 1 to 76%) in the skin samples analyzed in our study (with a median percentage of myeloid cells in the infiltrate of 20%). The “VAF/percentage of myeloid cells in the skin infiltrate” ratio was similar between *leukemia cutis* and our skin samples (p=0.265 using Mann-Whitney test), supporting that mutations detected in skin biopsies of our MDS/CMML patients were related to a significant clonal myeloid infiltrate.

## Discussion

This case-series study highlights that despite heterogeneous clinical phenotypes of cutaneous lesions associated with MDS/CMML, a significant clonal skin infiltrate of MDS/CMML cells is found in most cases.

Recently, it has been shown using FISH in four patients and NGS in one patient followed for MDS that cutaneous lesions infiltrated by immature myeloid non-blastic cells were clonally related to the myeloid malignancy, and some authors suggested that they should be classified as *myelodysplasia cutis* (15, 16). Similarly, studying skin biopsies of patients with SS in the setting of MDS/CMML or acute myeloid leukemia, Passet et al. found a clonal infiltrate on the biopsies of 4/4 patients with a neutrophilic-SS associated with MDS/CMML (14). Our results confirmed these findings as all four patients diagnosed as H-SS before NGS had a clonal infiltrate related to the MDS and highlighted that other histiocytic or neutrophilic cutaneous disorders such as neutrophilic pustules, adult-xanthogranuloma, and pyoderma gangrenosum may also be characterized by a clonal infiltrate that shares a common clonal progenitor with MDS/CMML. Most patients had shared common mutations between paired blood/marrow and skin samples. Indeed, half of patients had identical paired mutations in blood/marrow and skin lesions, whereas some patients had additional mutations in skin or blood/bone marrows (n=3 each). This could be explained by a dissociated clonal evolution between hematopoietic tissue and skin once the original clone has spread to the skin.

Caution must, however, be exercised when interpreting VAF, which must be correlated to the percentage of myeloid cells infiltrating the skin to determine its significance and rule out a blood contamination, as in patient 8. Low VAF can be hard to interpret as in patient 5, considered negative but who also had a low circulating clonal burden, the low skin VAF thus did not strictly rule out a specific cutaneous localization of the myelodysplastic syndrome in this case.

The clinical diversity observed in our study is consistent with previous data in MDS/CMML-associated inflammatory manifestations. Despite efforts to classify these SIADs, they sometimes remain unlabeled. Apart from trisomy 8 associated with Behçet’s-like disease, no specific chromosomal abnormality has been associated with a particular clinical phenotype until now (7). Interestingly, an adult-onset autoinflammatory disease associated to *UBA1* somatic mutations was recently described in 25 patients, 24% showing MDS and 88% having skin involvement with lesions such as nodules and plaques (compatible with neutrophilic dermatosis or cutaneous vasculitis), periorbital swelling, and severe cutaneous reaction to anakinra injection (21). In our study, we describe two new cases of VEXAS syndrome in patients with MDS associated to H-SS and extracutaneous symptoms. Furthermore, mutations in *TET2* and *DNMT3A* were the two most prevalent (four patients each) but are also among the most frequent mutations associated with MDS. On the contrary, mutations in *SF3B1*, found in approximately 20% patients with MDS, was only found in one patient with low cutaneous VAF in our study (22). Given the small number of patients and the clinical diversity, no mutation could be associated with a particular cutaneous phenotype, and whether some mutations are associated with particular skin lesions still needs to be clarified.

The demonstration of a common clonal progenitor between myeloid neoplasms and neutrophilic and H-SS (12, 14–16, 23) as well as clonal association between L-Group histiocytosis and MDS/CMML (18, 24) supports a role of blood cells in the development of extramedullary expression of MDS/CMML and enlightens the pathophysiology of these manifestations, what may guide treatment. Indeed, azacytidine seems to have a potential effect on MDS/CMML-associated SIADs in a retrospective study (25). In our series, hematological treatment proved at least partly efficient on skin lesions in five patients. NGS positivity did not seem associated with treatment response as the two non-responders had a cutaneous clonal infiltrate. Beyond extramedullary hematopoiesis, the upregulation of inflammatory pathways in the clonal cells could also explain the occurrence of skin lesions. Indeed, monocytes in CMML have highly pro-inflammatory features (26), and mutations commonly seen in MDS/CMML happen to be associated with higher inflammation (27). Interestingly, the case of a patient with CMML and CMML *cutis* was reported in an IL-10 pilot trial with marked improvement of skin lesions under treatment (28). The dysregulation of pro-inflammatory pathways could explain the efficacy of immunosuppressive treatments in some patients.

Our study has several limitations. First, we enrolled a limited number of patient due to the rarity of the condition studied and the enrollment in only three hospitals. Second, two patients did not have a concomitant blood/marrow and skin sampling, limiting the comparability of mutations in blood/marrow *versus* skin. Third, some data were collected retrospectively as patients were enrolled at the time skin lesions appeared. Fourth, concurrent treatment administration was frequent, limiting outcome interpretation. At last, our study design does not allow to assess the overall prevalence of cutaneous lesions among MDS/CMML patients and to assess differences between patients with or without cutaneous lesions in terms of molecular profile and prognosis.

Our data need further investigation including a genetic study of the other organs involved in SIADs associated with MDS/CMML and of the incidence of extramedullary clonal infiltration over survival.

## Data Availability Statement

The original contributions presented in the study are included in the article/supplementary material. Further inquiries can be directed to the corresponding author.

## Ethics Statement

Ethical review and approval was not required for the study on human participants in accordance with the local legislation and institutional requirements. The patients/participants provided their written informed consent to participate in this study. Written informed consent was obtained from the individual(s) for the publication of any potentially identifiable images or data included in this article.

## Author Contributions

GF: conceptualization, data curation, formal analysis, methodology, and writing original draft. PH, SGM, FC, and AM: conceptualization, methodology, supervision, validation, and writing review and editing. Other authors: writing review and editing. All authors contributed to the article and approved the submitted version.

## Conflict of Interest

The authors declare that the research was conducted in the absence of any commercial or financial relationships that could be construed as a potential conflict of interest.

## Publisher’s Note

All claims expressed in this article are solely those of the authors and do not necessarily represent those of their affiliated organizations, or those of the publisher, the editors and the reviewers. Any product that may be evaluated in this article, or claim that may be made by its manufacturer, is not guaranteed or endorsed by the publisher.
